# Bond Behavior of Historical Clay Bricks Strengthened with Steel Reinforced Polymers (SRP)

**DOI:** 10.3390/ma4030585

**Published:** 2011-03-21

**Authors:** Ernesto Grande, Maura Imbimbo, Elio Sacco

**Affiliations:** Department of Mechanics, Structures and Environments, University of Cassino—Italy/via G. Di Biasio 43, 03043, Cassino (FR), Italy; E-Mails: e.grande@unicas.it (E.G.); mimbimbo@unicas.it (M.I.)

**Keywords:** bond, masonry, SRP, historical bricks, fracture energy

## Abstract

In the strengthening interventions of past and historical masonry constructions, the non-standardized manufacture processes, the ageing and the damage of masonry units, could significantly affect the properties of the surfaces where strengthening materials are applied. This aspect requires particular care in evaluating the performance of externally bonded strengthening layers, especially with reference to the detachment mechanism. The bond response of old masonries could be very different from that occurring in new masonry units which are the ones generally considered in most of the bond tests available in technical literature. The aim of the present paper is the study of the bond behavior of historical clay bricks strengthened with steel reinforced polymers (SRP) materials. In particular, the results of an experimental study concerning new manufactured clay bricks and old bricks extracted from different historical masonry buildings are presented. The obtained results, particularly in terms of bond resistance, detachment mechanism and strain distributions, are discussed for the purpose of analyzing the peculiarities of the historical bricks in comparison with new manufactured ones. Some considerations on the efficacy of the theoretical formulations of the recent Italian code are also carried out.

## 1. Introduction

Masonry structures constitute an important part of existing buildings in several countries and, in many cases, they also represent the cultural heritage of these countries, which has to be preserved and protected. The degradation of materials due to the ageing of structures and the damage caused by past events, primarily earthquakes, has increased the vulnerability of these structures. Although several standards codes and documents contain specific indications about the strengthening interventions on existing masonry structures, one of the key aspects, in the case of cultural heritage, is to select strengthening solutions characterized by high effectiveness, low invasiveness and reversibility [1].

The strengthening interventions based on the use of fiber composite materials (FRP, Fiber Reinforced Plastic) certainly represents an attractive solution able to guarantee all the above remarked properties. In recent years, new composite materials have been developed for the purpose of improving their compatibility with the structural materials in civil constructions and reducing costs. Among the new generation of composite materials, there is a great relevance of unidirectional ultra high tensile steel sheets made of steel fibers, characterized by high tensile strength and reduced diameters and impregnated with epoxy resin, in the case of Steel Reinforced Polymer (SRP), or impregnated with mortars (cement or hydraulic) in the case of Steel Reinforced Grout (SRG). The steel filaments are braided into cords of various geometries and characterized by different values of strength and glued on a polypropylene scrim inorder to simplify installation procedures. The application of SRP/SRG materials seems to be very suitable to masonry structures, thanks to the possibility that these materials can be impregnated *in situ*, can be easily removed, and are extremely compatible with the substrate maintaining excellent mechanical properties. However, most of the applications have been done for concrete structures. Only quite recently, the use of SRP/SRG materials has been extended also to the strengthening of masonry structures; some examples are the interventions made in important Italian constructions, such as the Church of San Gaetano in Padua, the Museum Diocesano in Faenza, and the building of Contucci in Montepulciano [2,3].

A crucial point in the design and use of externally bonded reinforcement for the strengthening of masonry constructions is the bond mechanism between the reinforcement and masonry. Indeed, in recent years there has been an increasing interest in theoretical and experimental studies on bond behavior. However, most of these studies refer to the strengthening of new manufactured masonry units, which, in contrast to the old ones, are generally produced using a standardized manufacturing process, and are characterized by high compactness of clay and having very tough surfaces. Moreover, investigations have been developed for FRP strengthening materials; while very few studies refer to old bricks reinforced by SRP.

For this purpose, the present paper shows and discusses the results of experimental bond tests involving new and historical clay bricks strengthened by SRP. The obtained results have particularly evidenced the role that some parameters—such as the compressive strength, the porosity and the damage of the surface of bricks—play in the local and global bond behavior of historical clay bricks reinforced by SRP. On the basis of the obtained results, the efficacy of the theoretical formulations provided in the recent Italian code CNR-DT 200 [4] is also discussed.

The paper is composed of three main parts. The first part concerns the description of the specimens, the experimental setup and the preliminary tests for characterizing the bricks; the second part concerns the discussion of the bond tests results; the third part refers to the comparison between the experimental results and the theoretical formulations. A conclusive section is provided at the end of the paper.

## 2. Experimental Study

The experimental study presented in this paper consists of double shear push-pull tests performed on new and historical clay bricks strengthened with SRP materials. Additional tests are also provided in order to characterize the bricks. In particular, three series of five specimens, for a total of fifteen bricks, were tested for the bond experiments, and compressive tests were performed on cubic specimens extracted from the bricks.

The results obtained from the tests are presented in terms of bond resistance, detachment mechanism and strain distribution along the strengthening component. The compressive strength and the characteristics of the external surface of the bricks are also examined in order to analyze the obtained results.

### 2.1. Materials

#### 2.1.1. Clay Bricks

**Figure 1:**
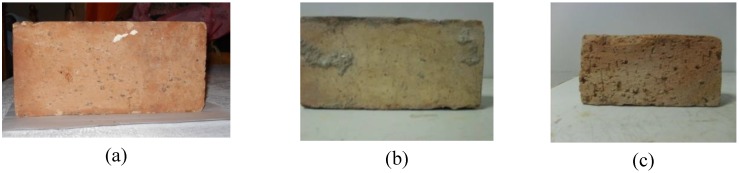
Examined bricks.

Three different types of clay bricks have been considered; they are denoted in the following as “new”, “old-1” and “old-2”:

- the “new” bricks, illustrated in Figure 1a, are produced in the south of Italy;
- the “old-1” bricks, illustrated in Figure 1b, were extracted from a warehouse built between 1920–1930 and located in the south of Italy (Teano, Caserta);
- the “old-2” bricks, illustrated in Figure 1c, were extracted from a construction adjacent to a convent, which was built between 1940–1950 in the center of Italy (Ceccano, Frosinone).

**Figure 2:**
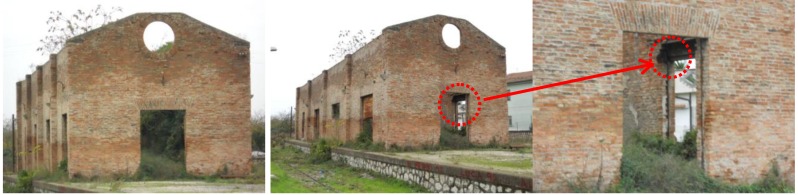
Warehouse (from which the old-1 bricks were extracted).

In particular, the warehouse is a one story building with rectangular dimensions about 50 × 9.0 m^2^ (see Figure 2). Both the external and the internal surfaces of the walls, composing the structure, are not covered by plaster and show a regular texture composed of a double layer of clay bricks. A timber roof was present on the structure until the years 1960–1970, when it completely collapsed due to the degradation of materials. The bricks used for the tests were extracted from the lateral wall near the door.

**Figure 3:**
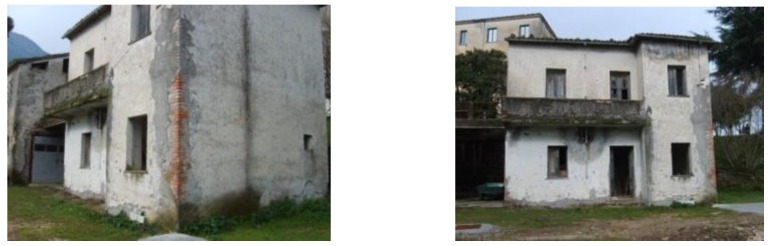
House next to the convent (from which the old-2 bricks were extracted).

The masonry construction, from which the “old-2” bricks were extracted, is a simple two-story house (see Figure 3) next to an important convent (“convent of Padri Passionisti” in Ceccano, Frosinone). In this case, the walls of the construction are composed of tuff blocks and clay bricks. In addition, according to a traditional rule of construction, clay bricks are also inserted along the corners of the building (see Figure 3) and around the doors and the windows. All the façades are covered in plaster and the examined bricks were extracted from the first story during a restoration intervention.

All the examined bricks have similar dimensions, about 120 × 55 × 250 mm, although there are several differences, particularly in terms of characteristics of exterior surfaces. Whilst “new” bricks show compact surfaces with regular and uniform porosities for all the examined specimens, the “old-1” and “old-2’ bricks present different properties also in comparison to the “new” bricks. The “old-1” bricks present compact and tough surfaces with porosity lower than the “new” bricks and, in some cases, with macro-irregularities due to the presence of several proturbances affecting the surface. The “old-2” bricks present uniform characteristics in terms of surface configuration with no macro-irregularities. However, in comparison to “new” bricks, they are characterized by greater and non-uniform porosity, lower compactness and, also, less tough surfaces. The measurement of these properties is quite complex, in particular for the porosity and the compactness which generally involve only a limited depth of the brick surface. It is worth noting that although the differences in terms of porosities were evident, old and new bricks are characterized by similar weights.

All the above aspects play an important role in the bond behavior since they affect the adherence between the support and the strengthening system and, consequently, the interaction process in terms of the stress transfer mechanism.

**Figure 4:**
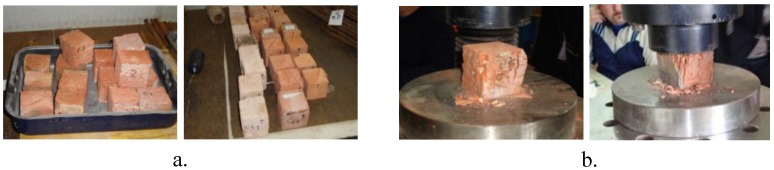
(**a**) Specimens extracted by cutting the bricks used for bond tests; (**b**) compressive tests.

Another important parameter analyzed in the investigation is the compressive strength of the bricks which has been evaluated by performing compressive tests, by means of the universal testing machine (Galdabini SUN60) on cubic specimens of dimensions equal to 50 mm. In particular, in the case of new bricks, the compressive test was performed on six cubic specimens all extracted from a brick which has not been used for the bond tests and the results are reported in Table 1. In the case of old bricks, the compressive tests were carried out on cubic specimens extracted from each brick used for bond tests. In particular, after the bond tests, the strengthening portion has been removed from the bricks, the surface of the bricks has been leveled by removing the adhesive residues and six or eight cubic specimens have been obtained by cutting the bricks (Figure 4). Before carrying out the compressive tests, each specimen has been dried in an oven and weighed by using an electronic balance. The results obtained for old bricks are summarized in Table 2.

**Table 1:** Compressive test results (new bricks).

cubic specimen	compressive strength [MPa]
1	41.5
2	35.7
3	40.6
4	34.7
5	36.6
6	41.7
**average value**	**38.5**

**Table 2:** Compressive test results (old bricks) and bond tests results (new and old bricks).

Type	Specimen	Average compressive strength [MPa]	Bond resistance [N]	Debonding mechanism
“new”	S1	See Table 1a	10127	A
	S2		13354.5	A
	S3		11490	A
	S4		10627	A
	S5		14.615	A
	**Average**	**38.5**	**12043**	
”old-1”	S1	34.69	10573	A
	S2	30.18	10014.5	A
	S3	32.13	8342	A-B
	S4	34.74	5141	C
	S5	19.75	5689	C
	**Average**	**30.3**	**7952**	
”old-2”	S1	27.29	5908	B
	S2	22.29	8437.5	A-B
	S3	34.74	4805.5	B
	S4	17.37	7007	A-B
	S5	13.63	5113	B
	**Average**	**23.1**	**6254**	
Legend:	**A:** removal of a deep portion of the support material at the end of the reinforcement together with a thin layer of the support material along the other part of the strip **B:** detachment of a uniform and thin layer of the support material along the strip **C:** detachment of the adhesive **A-B:** combined mechanism			

#### 2.1.2. Strengthening System

For all the specimens, steel reinforced polymer strips, 25 mm wide, have been used. In particular, 3X2-B 12-12-500 hardwire (medium density) steel reinforced polymer has been applied on the brick surface by using a bi-component epoxy resin (Betontex, RC02-LC202). The main properties of the strengthening system, supplied by the producer (TEC.INN-srl, Italy) are reported in Table 3.

**Table 3:** Properties of the strengthening system.

FIDSTEEL 3X2-B-12-500 HARDWIRE	
Tensile strength of strip	3070 MPa
Ultimate deformation of strip	1.60%
Elastic modulus	190 GPa
Equivalent thickness	0.227 mm
**Betontex, RC02-LC202**	
Tensile strength	35 MPa
Ultimate deformation	>2.8%
Elastic modulus	>2.5 GPa

### 2.2. Specimens and Setup

**Figure 5:**
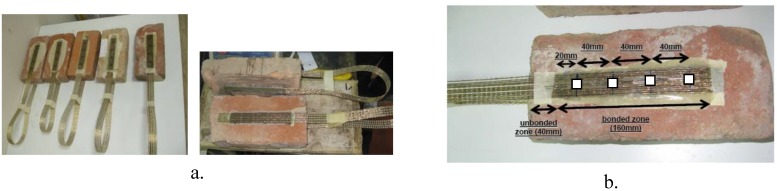
(**a**) Specimens’ configuration; (**b)** Strain gauges positions.

Five specimens for each type of brick have been tested in the bond experiments (Table 1). In particular, all specimens have been prepared according to a standardized procedure proposed by the RILEM committee (RILEM technical committee 223-MSC) and considering the configuration shown in Figure 5. A single strip, 25 mm wide, has been bonded on two opposite surfaces of the brick leaving an un-bonded zone of 40 mm and a bonded zone of 160 mm. For all types of the examined bricks, two specimens have been equipped with four strain gauges applied on the strengthening portion according to the configuration shown in Figure 5. For the other specimens, only the bond resistance and the detachment mechanism have been evaluated from the bond tests.

**Figure 6:**
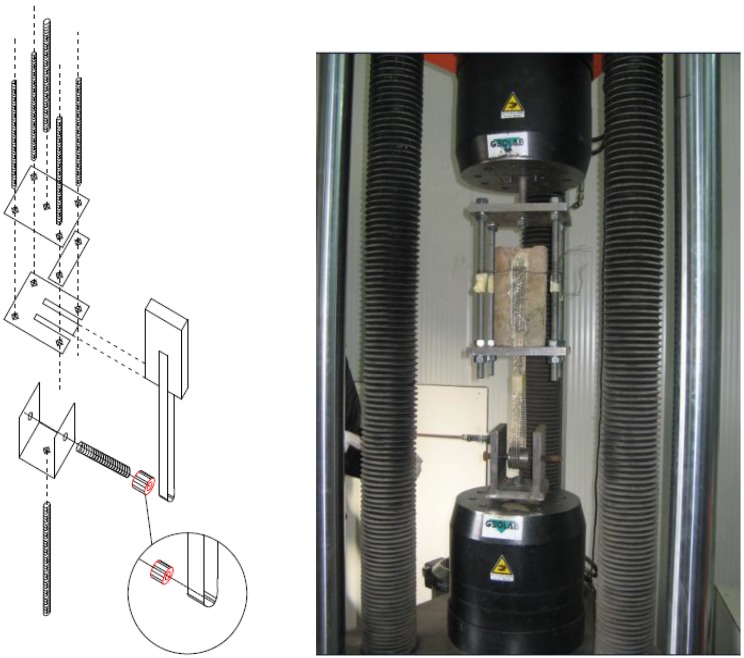
Test setup.

Bond tests have been performed by using the special device illustrated in Figure 6; the proposed test setup allows carrying out bond tests by simply using a universal testing machine present in several laboratories. In particular, the same machine used for performing the compressive tests has been adopted for the bond tests. The device consists of two main components: a steel frame made of two steel plates connected by four bars, and an additional system which consists of a steel roller for pulling the reinforcement. Other authors have also used a similar device for performing bond tests [5,6,7,8,9].

## 3. Results

**Figure 7:**
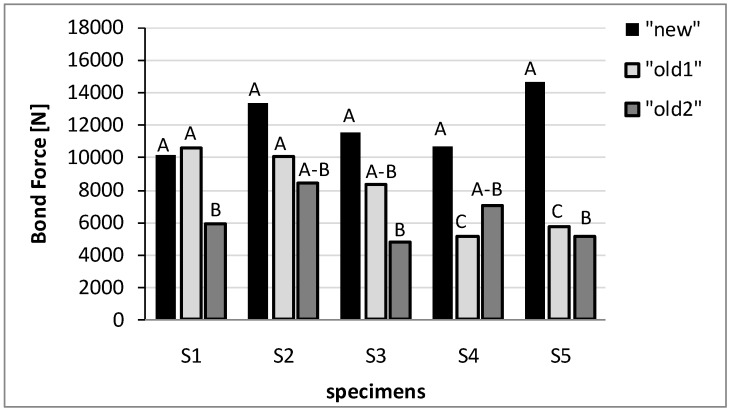
Bond resistance and detachment mechanism.

**Figure 8:**
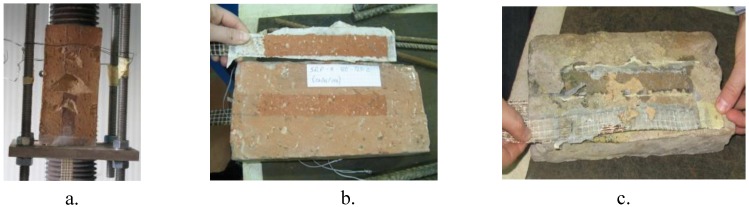
Detachment mechanisms: **a**. mechanism A; **b**. mechanism B; **c**. mechanism C.

The first results analyzed from the bond tests were the bond resistance and the detachment mechanisms. These data are reported in Table 1 where the bond resistance refers to one half of the bond force in order to take into account the resistance of a single strip. The same data are also plotted in Figure 7. The detachment mechanisms are shown in Figure 8.

Figure 7 shows that the “new” bricks are characterized by similar values of bond resistance, varying between 10.1 kN to 14.6 kN, and the same type of detachment mechanism; in fact, in this case, the detachment is characterized by the removal of a deep portion of the support material at the end of the reinforcement together with a thin layer of the support material along the other part of the strip (mechanism A, Figure 8).

In the case of “old-1” bricks, Figure 7 shows that the examined specimens are characterized by different values of bond resistance and different types of detachment mechanisms. In fact, specimens S1 and S2 showed similar values of bond resistance, about 10 kN, and the same bond mechanism (mechanism A) which also characterizes the “new” bricks. However, specimen S3 collapsed at a lower value of bond resistance, equal to 8.3 kN, with a mixed detachment mechanism involving mechanism A (but with a reduced depth of the removed support material) and, also, mechanism B which consists of a detachment of a uniform and thin layer of the support material along the strengthening portion (see Figure 8).

Specimens S4 and S5 showed the lowest values of bond resistance (less than the 50% of the maximum value of the bond resistance of the others specimens) and a different debonding mechanism, characterized by the detachment of the adhesive (mechanism C, Figure 8). As observed in the previous section, these two specimens are characterized by irregular surfaces with significant irregularities and proturbances.

In the case of the “old-2” bricks, specimens S1, S3, S5 are characterized by low values of bond resistance varying from 4.8 kN to 5.9 kN and by the same type of debonding mechanism, consisting of the detachment of a uniform and thin layer of support material along the whole length of the strengthening portion (mechanism B). Specimens S2 and S4 showed greater values of bond resistance and a mixed debonding mechanism, which involves both mechanism A and mechanism B.

The bond tests also provided, for some specimens (“new”-55, “old-1”-S5, “old-2”-S3), the measurements of the strain distribution.

**Figure 9:**
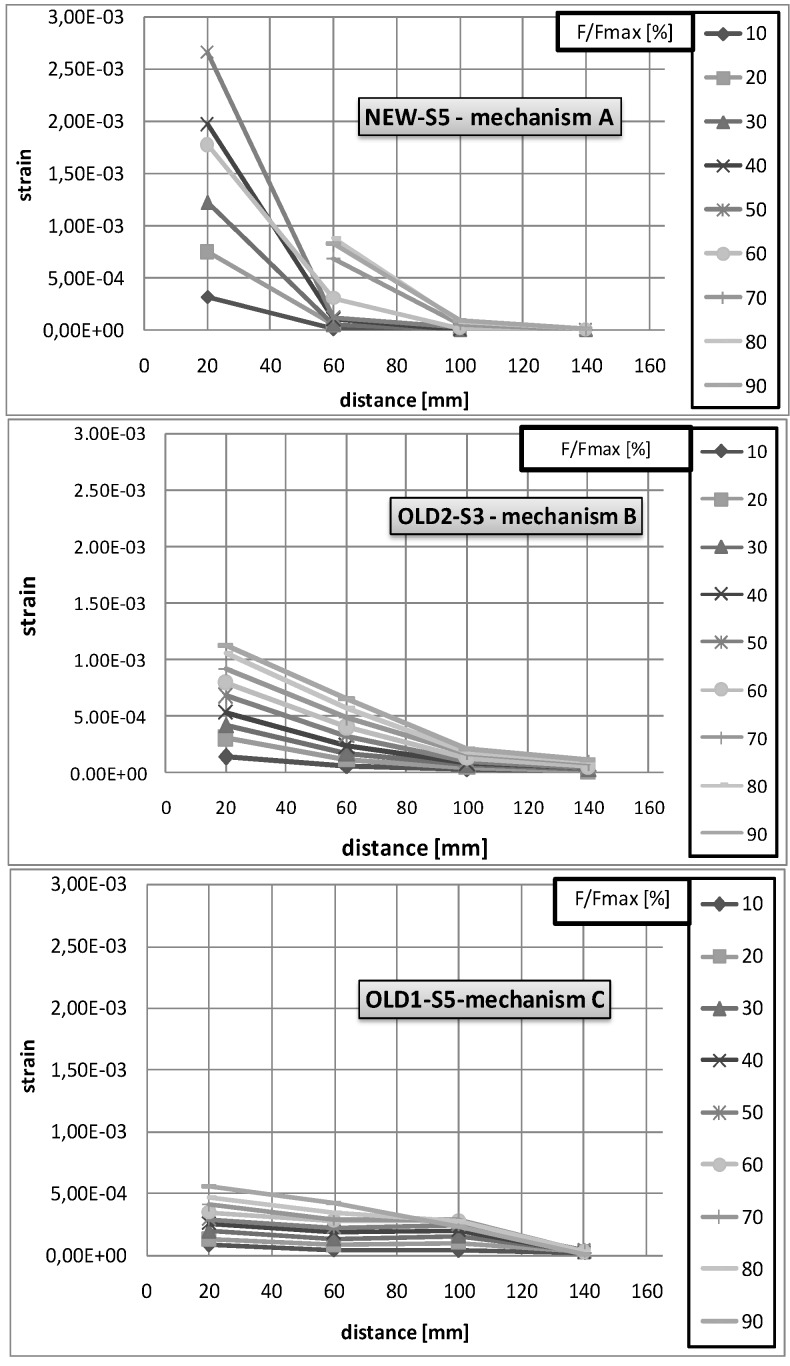
Strain distributions.

In the case of “new” bricks, whose failure occurs with mechanism A, the measured strain distribution is reported for different load levels, in Figure 9. The figure shows a concentration of strains in the initial portion of the strengthening portion until the load reaches the 80% of bond resistance when the strain concentration becomes lower and moves toward the central part of the strengthening portion.

In the same figure, the strain distributions measured in “old-1” and “old-2” bricks, which collapse in mechanisms B and C respectively, are also reported. The figure shows that for the brick with mechanism C, the strain distribution is uniform along the strip. However, for the brick with mechanism B, the strain distribution is somehow intermediate between those occurring in mechanism B and C; in fact, the strain concentration is less evident than that which occurred in mechanism A and is placed in the centre of the strengthening portion.

From Figure 9 it is also evident that the maximum values of the strain occurred using mechanism A, whilst the minor ones occurred using mechanism C.

## 4. Discussion and Remarks

The experimental tests have so far evidenced a different bond behavior of historical clay bricks in comparison to new bricks. In particular, old bricks have shown a major variability of bond resistance and of the detachment mechanism. These differences can be correlated to some characteristics of old bricks and, primarily, the compressive strength and the properties of the exterior surfaces where the strengthening is applied.

### 4.1. Compressive Strength

**Figure 10:**
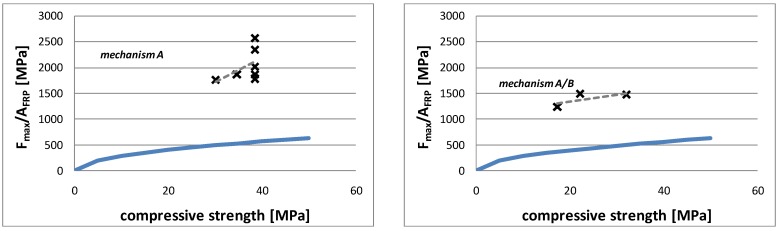

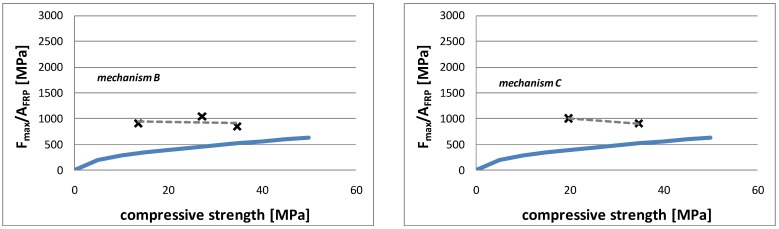
Bond resistance *vs*. compressive strength.

In order to analyze the influence of the compressive strength, the values of the bond resistance, divided by the cross section area of the FRP *versus* the compressive strength, are reported in Figure 10, for each specimen. The data, represented by cross marks, are collected on the basis of the collapse mechanism occurring in the specimens. The tendency line (dotted line) is also shown in the same figure. The first plot refers to the specimens using mechanism A, which involves the removal of a deep portion of the support material; while the second plot relates to mechanism A/B, which involves the removal of a deep portion of the brick material at the end of the strip and the detachment of a thin layer of the brick material along the remaining zone of the strip. From the plots, the influence of the support’s compressive strength on the experimental values of the bond strength of specimens is evident. This result is in agreement with the type of observed failure modes of specimens in which the core material of the brick is responsible for the compressive strength of the support. In the case of mechanism B, which involves the removal of a thin and uniform layer of the brick material along the whole length of the strip, the compressive strength of the support does not influence the bond strength of the specimens. This result is also in agreement with the observed failure mode; in this case, the bond strength depends only on the shear strength of the material composing the external layer surface of the brick, without involving the inner material of the support. Finally, by examining the two specimens collapsing with mechanism C, which involves the detachment of the adhesive layer, it is evident that, also in this case, the compressive strength of the support does not influence the bond strength.

### 4.2. External Surface

Regarding the properties of the external surface of the bricks, it has been observed that, in the case of new bricks, the external surface is characterized by uniform porosities and a good level of compactness (absence of external layers with mechanical properties lower than those characterizing the core material of the brick), factors which guarantee a good adherence of the reinforcement (due to the penetration of the adhesive) and a high resistance of the support surface with respect to the detachment. On the other hand, old bricks are characterized, in several cases, by macro-irregularities (specimens S4, S5 of “old-1” bricks) which do not guarantee an adequate level of adherence of the strengthening to the support, or by weak surfaces with high porosity, which guarantee a good adherence but lead to a low bond strength of the exterior support material (specimens S1, S3, S5 of the “old-2” bricks).

These differences are also confirmed by the strain distributions which showed a variability of the stiffness of the interface layer between the FRP and the masonry support; this variability is due to the mechanical properties of the support and the adhesive material but, also, depends on the characteristics of the external surface of the bricks (porosity and regularity). In fact, similar detachment mechanisms have been observed for bricks characterized by different values of the compressive strength and similar characteristics of the external surfaces (see for instance “old-2”-s1 and “old-2”-s3) and, conversely, different detachment mechanisms have occurred for bricks characterized by similar values of compressive strength and different features of external surfaces (see for instance “old-2”-s1 and “old-2”-s3).

It is clear that these aspects must be accounted for in theoretical models, in order to also capture the bonding behavior in the case of historical bricks.

## 5. Theoretical Formulation

The results obtained from the experimental tests on the different types of examined bricks have evidenced so far the role of different factors on the bond behavior of clay bricks strengthened by SRP materials.

On the basis of these experimental results, it would be worthy to verify the efficacy of the theoretical formulation provided by the Italian code CNR-DT 200 [4] and the adopted assumptions.

In particular, the CNR-DT200, according to other codes which refer to concrete supports [10,11] suggests evaluating the bond strength *f_fdd_* of masonry elements strengthened by FRP materials through the following equation:

(1.a) $$f_{fdd}=\frac{1}{\gamma_{fd}\sqrt{\gamma_{M}}}\sqrt{\frac{2\cdot E_{f}\cdot \Gamma_{f}}{t_{f}}}\;(if\;L_{b}\geq L_{e})$$

(1.b) $$f_{fdd,rid}=f_{fdd}\frac{L_{b}}{L_{e}}\left(2-\frac{L_{b}}{L_{e}}\right)\;(if\;L_{b}<L_{e})$$

where $\gamma_{fd}$ is a safety factor that takes into account the modality of the application of the reinforcement system; $\gamma_{M}$ is a safety factor concerning the masonry material; $E_{f}$ and $t_{f}$ are respectively the Young’s modulus and the thickness of the strengthening; $\Gamma_{f}$ is the fracture energy; $f_{fdd,rid}$ is the reduced value of the design bond strength; $L_{b}$ is the bond length of FRP element; $L_{e}$ refers to the optimal bond length of FRP corresponding to the minimal bond length able to carry the maximum anchorage force, which can be deduced through the following equation also reported in the CNR-DT200:

(2) $$L_{e}={\left[E_{f}\cdot t_{f}/2f_{mtm}\right]}^{0.5}$$

where $f_{mtm}$ the masonry average tensile strength.

A further indication contained in the CNR-DT200 concerns the evaluation of the fracture energy. With regard to this last parameter, the CNR-DT200 gives the following relation based on the compressive strength $f_{mk}$ and the tensile strength $f_{mtm}$ of the support:

(3) $$\Gamma_{f}=c_{1}\sqrt{f_{mk}\cdot f_{mtm}}$$

where $c_{1}$ is an experimental coefficient which, in the case of masonry supports, is suggested to be assumed equal to 0.015 if experimental results on the fracture energy are not available.

### 5.1. Remarks on the Coefficient *c_1_*

The evaluation of the fracture energy is generally very difficult to obtain because of the rapid development of the detachment phenomenon during the tests. In fact, also in the tests discussed in this paper, it was not possible to evaluate the fracture energy despite the fact that the tests were conducted by a displacement control procedure with low velocities equal to 0.3 mm/min. In addition, this datum is generally not available when a preliminary design of the strengthening system is carried out. This implies that also the experimental coefficient $c_{1}$ is generally assumed by adopting the code indications.

On the basis of the above considerations, it is worth analyzing if the value 0.015 suggested by the code can be considered valid also for historical bricks strengthened by SRP. To this purpose, considering Equations (1.a) and (3), assuming an unitary value for the safety coefficients and setting the tensile strength equal to $0.10\cdot f_{mk}$ as suggested by the code, the coefficient $c_{1}$ can be evaluated through the following formulation:

(4) $$c_{1}=\frac{f_{f,\exp}^{2}\cdot \frac{t_{FRP}}{2E_{FRP}}}{\sqrt{f_{mk}\cdot f_{mtm}}}$$

where $f_{f,\exp}$ is the experimental value of the bond strength. The bond strength is defined as the bond force resulting from the tests divided by the cross section area of the FRP strip.

The values of the coefficients $c_{1}$ deduced from Equation (4) are reported in Table 4.

**Table 4:** Experimental values of the coefficient *c1* (Equation 4).

Specimen / debonding mechanism	Coefficient *c_1_*
“new” / mechanism A	0.230
“old-1” / mechanism A	0.190
“old-2” / mechanism B	0.076
“old-1” / mechanism C	0.070
“old-1”, “old-2” / mechanism A-B	0.160

The comparison between the values obtained for new bricks with those obtained for old bricks, suggests the following observations:

- old bricks (“old-1”/mechanism A and “old-1”, “old-2”/mechanism A-B), characterized by values of the compressive strength similar to new bricks (differences less than 20%) and by regular surfaces with a uniform distribution of porosities, show a reduction of the average value of the coefficient $c_{1}$ approximately equal to 15%;
- old bricks (“old-2”/mechanism B and “old-1” mechanism C), characterized by irregular surfaces or by values of compressive strength less than the values characterizing new bricks (differences greater than 40%), show a reduction of the average value of the coefficient $c_{1}$ greater than 65%. In all the above cases, the obtained values of c_1_ are significantly greater than the value proposed by the code.

### 5.2. Influence of the Compressive Strength

The theoretical values of the bond strength evaluated by Equation 1.a, assuming the safety factors equal to 1, are charted as a continuous line in Figure 10, as previously discussed in Section 4.

The first two plots, referring to mechanism A and A/B, show that the theoretical formulation is able to represent the dependency of the support’s compressive strength on the experimental values of the bond strength in accordance with the experimental results. However, in the case of mechanism A, the experimental values and the dependency of the bond strength on the compressive strength are much higher than the results provided by the theoretical model. Moreover, the results carried out for new bricks show a great variability which could probably be due to the fact that the compressive strength was not directly evaluated on the specimens used for bond tests. On the other hand, in the case of mechanism A/B, the theoretical formulation leads to a better estimation of the dependency of the bond strength on the compressive strength of the support.

In the case of mechanism B, and also mechanism C, the theoretical values are in good agreement with the experimental values, although, in this case, the dependency on the compressive strength given by the theoretical formulation is not confirmed by the experimental results.

### 5.3. Optimal Bond Length

**Figure 11:**
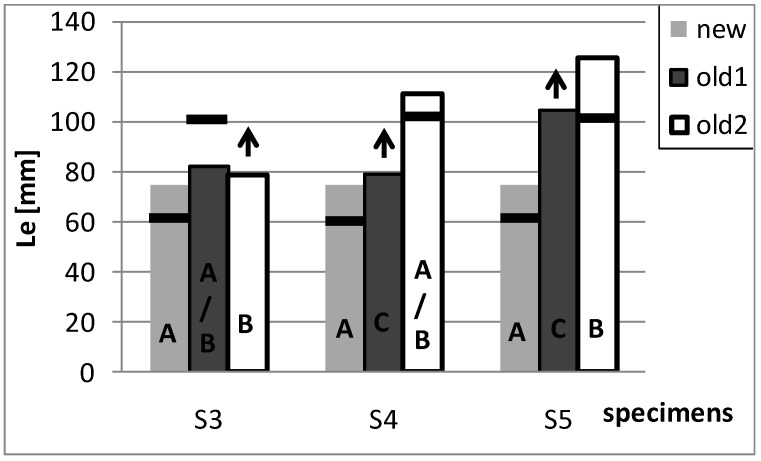
Optimal bond length.

A further parameter examined in light of the theoretical formulation provided by the code, concerns the optimal bond length *L_e_* which is particularly influenced by the characteristics of the support. In Figure 11 the comparison between the theoretical values of the optimal bond length *L_e_* (vertical bars) and the experimental values (horizontal segments) is reported. In particular, the experimental values of *L_e_*, deduced on the basis of strain-gauges measurements, has been assumed equal to the distance between the section where the maximum strain value is attained and the section where a strain value not greater than 5% of the maximum strain is attained. On the contrary, the arrow symbols indicate the cases where the minimum measured value of strain is greater than 5% of the maximum strain: this means that the experimental optimal bond length is greater than the length of SRP.

From the plot, it is evident that all the “new” specimens (characterized by mechanism A) show a theoretical value of *L_e_* greater than the experimental ones, whilst the contrary occurs for the “old-1” bricks and the specimen S3 of the “old-2” bricks. This result is probably due to the fact that the porosity and the regularity of the surface of the “new” bricks guarantees an excellent anchorage level of the reinforcement to the support; whereas the irregular surface and the minor porosity of the “old-1” bricks, together with the different failure mechanisms, lead to a reduction of the anchorage effect of the SRP. The above observations, again, underline the role of the support’s surface on the bond behavior and, also, emphasize the different behavior of the historical bricks in comparison to new manufactured elements as already discussed in the previous section.

## 6. Conclusions

In this paper the results of bond tests conducted on new manufactured and old bricks have been discussed.

The different bond behavior that emerged from the tests has been examined on the basis of the different characteristics of the bricks, particularly in terms of the compressive strength and the properties of the exterior surfaces where the strengthening is applied.

The obtained results have clearly shown the important role of both characteristics. In particular, the tests showed that regular surfaces with uniform porosities distribution, which are commonly found in new bricks and some old bricks, lead to a good level of adherence between the strengthening system and the support, and are characterized by a debonding mechanism which involves the detachment of the support material. In this case the compressive strength of the support material plays an important role in the bond resistance and, thus, stiff surfaces and bricks materials characterized by high values of compressive strength contribute to increase the bond resistance. On the other hand, bricks characterized by macro-irregularities, such as some types of old bricks, do not guarantee an adequate level of adherence of the strengthening to the support and are characterized by a debonding mechanism which involves the detachment of the adhesive with the lowest values of bond resistance. Finally, bricks with weak surfaces but high porosity, such as others types of old bricks, can guarantee a good adherence, but show a low bond strength of the exterior support material which leads to a debonding mechanism involving the detachment of a thin layer of the support material. In both these last cases the compressive strength lightly affects the debonding resistance.

It is evident that these aspects have to be particularly considered when a preliminary estimation of the bond resistance is carried out by using the design formulations contained in the codes provided or when referring to results deduced with reference to the same type of masonry units but with different characteristics.

Finally, it is important to note that, although this study has shown important peculiarities concerning the strengthening of historical constructions with innovative composites materials, further aspects and additional information can be developed by considering other masonry materials and a greater number of experimental tests. Nevertheless, as emphasized in this paper, it is very important to improve the actual studies on the discussed problem and the relevant code provisions.
